# The Loop of the TPR1 Subdomain of Phi29 DNA Polymerase Plays a Pivotal Role in Primer-Terminus Stabilization at the Polymerization Active Site

**DOI:** 10.3390/biom9110648

**Published:** 2019-10-24

**Authors:** Alicia del Prado, Eugenia Santos, José M. Lázaro, Margarita Salas, Miguel de Vega

**Affiliations:** Centro de Biología Molecular Severo Ochoa (Consejo Superior de Investigaciones Científicas-Universidad Autónoma de Madrid), Universidad Autónoma, Cantoblanco, 28049 Madrid, Spain; adelprado@cbm.csic.es (A.d.P.); mes_delrio@hotmail.com (E.S.); jmlazaro73@gmail.com (J.M.L.); mdevega@cbm.csic.es (M.d.V.)

**Keywords:** DNA polymerase, replication, protein-priming, terminal protein

## Abstract

Bacteriophage Phi29 DNA polymerase belongs to the protein-primed subgroup of family B DNA polymerases that use a terminal protein (TP) as a primer to initiate genome replication. The resolution of the crystallographic structure showed that it consists of an N-terminal domain with the exonuclease activity and a C-terminal polymerization domain. It also has two subdomains specific of the protein-primed DNA polymerases; the TP Regions 1 (TPR1) that interacts with TP and DNA, and 2 (TPR2), that couples both processivity and strand displacement to the enzyme. The superimposition of the structures of the apo polymerase and the polymerase in the polymerase/TP heterodimer shows that the structural changes are restricted almost to the TPR1 loop (residues 304–314). In order to study the role of this loop in binding the DNA and the TP, we changed the residues Arg306, Arg308, Phe309, Tyr310, and Lys311 into alanine, and also made the deletion mutant Δ6 lacking residues Arg306–Lys311. The results show a defective TP binding capacity in mutants R306A, F309A, Y310A, and Δ6. The additional impaired primer-terminus stabilization at the polymerization active site in mutants Y310A and Δ6 allows us to propose a role for the Phi29 DNA polymerase TPR1 loop in the proper positioning of the DNA and TP-priming 3’-OH termini at the preinsertion site of the polymerase to enable efficient initiation and further elongation steps during Phi29 TP-DNA replication.

## 1. Introduction

Most DNA polymerases are unable to start de novo DNA synthesis, as the presence of a 3’-OH group is necessary, which is generally provided by a short RNA or DNA molecule. In addition, they synthesize DNA exclusively in the 5’–3’ direction [1], causing the so-called end-replication problem [2]. Thus, there is a short region of unreplicated single-stranded DNA (ssDNA) that will remain at the end of the chromosome and would lead to a continuous shortening when the most terminal primer is removed. Organisms containing linear genomes have developed different strategies to overcome this problem. Several prokaryotic and eukaryotic viruses, as well as linear plasmids from bacteria, fungi, higher plants, and *Streptomyces* ssp. have solved the end replication problem by using a protein, called terminal protein (TP) that provides the priming OH group, to initiate DNA replication becoming covalently linked to the 5’ end of the nascent DNA [3,4,5].

Bacteriophage Phi29 infects *Bacillus subtilis* and has become the paradigm in the study of the protein-primed replication. It has a linear double-stranded DNA (dsDNA) with a TP covalently linked at each 5’ DNA end (TP-DNA). A heterodimer formed by the DNA polymerase and a free TP molecule (primer TP) recognize the replication origin [6]. Once bound to the replication origin, the DNA polymerase of the heterodimer catalyzes the incorporation of the initiating dAMP onto the OH group of the TP priming residue, Ser232 [3], in a reaction directed by the 3’ penultimate dTMP of the template strand. Then, by means of the so-called sliding-back mechanism, the initiation product (TP-dAMP) translocates one position backward to recover the information of the 3’ terminal dTMP [7]. This mechanism requires a terminal repetition of at least two nucleotides. Once the initiation has taken place, there is not an immediate dissociation of the heterodimer. A transition stage has been described between the initiation with the TP and the elongation of the DNA, in which the DNA polymerase synthesizes the first nine nucleotides, and after the incorporation of the 10th nucleotide, the DNA polymerase dissociates from the TP [8]. Then, the same DNA polymerase catalyzes processive chain elongation via a strand displacement mechanism to completely replicate the Phi29 TP-DNA molecule [9].

Phi29 DNA polymerase is the only member of the protein-primed subgroup of DNA polymerases in which the structure has been solved [10] and is composed of the N-terminal 3’–5’ exonuclease domain (residues 1–189) and the C-terminal polymerization one (residues 190–572). The latter domain contains the residues responsible for DNA synthesis and is subdivided into the universally conserved subdomains; palm, thumb, and fingers. There are also two insertions specifically present in the protein-primed DNA polymerases subgroup called Terminal Protein Regions 1 (TPR1) and 2 (TPR2). The TPR1 subdomain is involved in the interaction with the TP and the DNA [11,12,13], and the TPR2 confers both processivity and strand displacement capacity [14]. The crystallographic structure of the heterodimer shows that the TP has an N-terminal domain responsible for the localization of the TP in the bacterial nucleoid [15]; an intermediate domain that interacts with the TPR1 subdomain of the DNA polymerase, this interaction being responsible for both the specificity between the DNA polymerase and the TP and the maintenance of the heterodimer stability; and a C-terminal priming domain that occupies the DNA binding groove of the DNA polymerase to allow the proper placement of the TP priming residue Ser232 at the preinsertion site to prime the initiation of replication [16].

**Figure 1:**
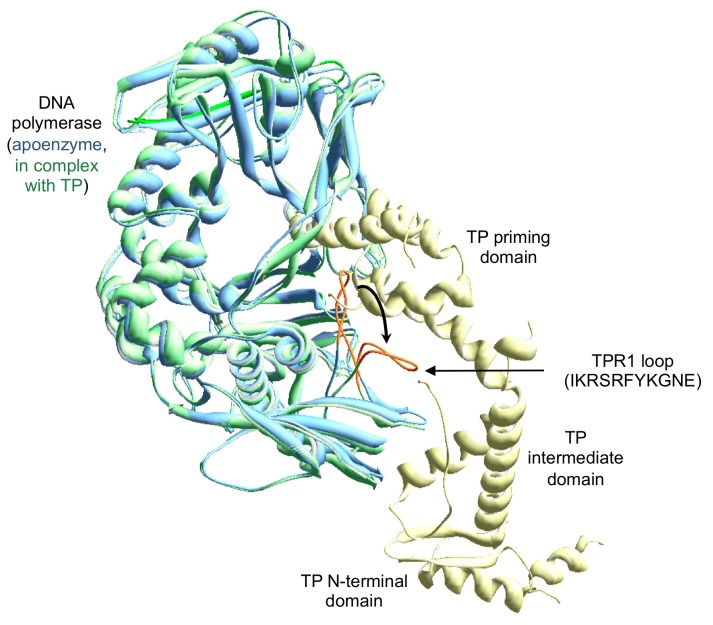
Movement of the Phi29 DNA polymerase TPR1 loop to allow the placement of the terminal protein (TP) priming domain. The figure was made by superimposing the structure of the DNA polymerase complexed with the TP [16] (PDB (Protein Data Base) code 2EX3) on the apo enzyme [10] (PDB code 1XHX). The DNA polymerase from the heterodimer and the apo enzyme are colored in green and blue, respectively. TP is colored in pale yellow. The TPR1 loop is colored in orange, and its sequence (from residue 304 to 314) is indicated.

The superposition of the crystallographic structure of the apo polymerase and the DNA polymerase forming a complex with the TP showed that in the main the overall structure of the enzyme remains mostly unchanged with the only exception of the conformational changes restricted to the TPR1 residues 304–314 [16]. In the apo enzyme structure, these residues of TPR1 form a loop with varied conformations with a substantial degree of flexibility. The crystallographic structures of the DNA polymerase/TP heterodimer and DNA polymerase ternary complex show that the access of both the DNA and TP priming domain to the catalytic site requires the previous bending of this loop [16], which acquires a platform shape on which both the DNA and the TP would slide as the DNA is synthesized (see Figure 1). Biochemical studies with the independently expressed TP-priming and intermediate domains demonstrated that such a movement is dependent on the previous interaction between the DNA polymerase TPR1 subdomain and the TP-intermediate domain [12]. In order to know the importance of this TPR1 loop in the interaction with the DNA and the TP, here, we have changed the electropositive residues (Arg306, Arg308, and Lys311) and the aromatic ones (Phe309 and Tyr310) of the loop to alanine, as well as obtained the deletion mutant Δ6 lacking residues 306–311.

The biochemical analysis of the mutant derivatives allows us to propose a role of this TPR1 loop, not only in primer-terminus stabilization but also in the interaction with the TP primer, specifically through residues Arg306 and Tyr310, as will be discussed.

## 2. Materials and Methods

### 2.1. Nucleotides and DNAs

Unlabeled nucleotides were supplied by GE Healthcare (Buckinghamshire, UK), [α-^32^P] dATP (3000 Ci/mmol) and [γ-^32^P]ATP (3000 Ci/mmol) were supplied by Perkin Elmer (Boston, MA, USA). Oligonucleotides were obtained from Invitrogen (Waltham, MD, USA).

Oligonucleotide sp1 (15mer) (5’-GATCACAGTGAGTAC) was purified electrophoretically on 7 M urea-20% polyacrylamide gel and 5’-labeled with [γ-^32^P]ATP and phage T4 polynucleotide kinase. Labeled oligonucleotide 15mer was hybridized to oligonucleotide sp1c+6 (21mer) (5’-TCTATTGTACTCACTGTGATC) and sp1c+13 (5’ AGAAGTGTATCTGGTACTCACTGTGATC) in the presence of 0.2 M NaCl and 50 mM Tris-HCl, pH 7.5, resulting in primer/template structures.

### 2.2. Site-Directed Mutagenesis of Phi29 DNA Polymerase and Terminal Protein

DNA polymerase mutants were obtained using the QuikChange site-directed mutagenesis kit provided by Agilent Technologies (San Diego, CA, USA), using plasmid pJLPM [17] as a template containing the viral gene 2 that encodes the wild-type Phi29 DNA polymerase, or plasmid pT7-3 that harbors the Phi29 DNA polymerase exonuclease deficient mutant D12A/D66A [18]. The presence of the desired mutations, as well as the absence of additional ones, was determined by sequencing the entire gene. DNA polymerase mutants were expressed in *Escherichia coli* BL21(DE3) cells and further purified essentially the same as described for the wild-type DNA polymerase [19]. The TP gene was cloned into the plasmid pT7.3. It was purified essentially the same as described in [20]

### 2.3. Polymerase/3’–5’ Exonuclease (Pol/Exo) Coupled Assay

The primer/template molecule sp1/sp1c+6 (15mer/21mer) contains a 6 nt 5’-protruding end, and therefore, the primer strand can be used as substrate for DNA-dependent DNA polymerization and also for the exonuclease activity. The incubation mixture contained, in 12.5 µL, 50 mM Tris–HCl, pH 7.5, 10 mM MgCl_2_, 1 mM dithiothreitol (DTT), 4% (*v/v*) glycerol, 0.1 mg/mL bovine serum albumin (BSA), 1.2 nM of 5’-labeled sp1/sp1c+6 substrate, 30 nM of wild-type or mutant Phi29 DNA polymerase, and the indicated increasing concentrations of the four deoxynucleoside triphosphates (dNTPs). After incubation for 5 min at 25 °C, the reaction was stopped by adding ethylendiaminetetraacetic acid (EDTA) up to a final concentration of 10 mM. Samples were analyzed by electrophoresis in 7 M urea-20% polyacrylamide gels and autoradiography. Polymerization or 3’–5’ exonucleolysis is detected as an increase or decrease, respectively, in the size (15mer) of the 5’-labeled primer.

### 2.4. Hydrolysis of p-Nitrophenol-Thymidine Monophosphate

The incubation mixture contained, in a volume of 300 µL, 50 mM Tris-HCl, pH 8.0, 150 mM NaCl, 1 mM DTT, 1 mM MnCl_2_, 3 mM of *p*-nitrophenol-TMP (*p*NP-TMP) dissolved in 50 mM Tris-HCl, pH 8.0 and 150 mM NaCl, and 500 nM of either the wild-type or the indicated mutant Phi29 DNA polymerase. Hydrolysis was evaluated by monitoring *p*-nitrophenol production at 420 nm with a Hitachi U-200 spectrophotometer at 25 °C, essentially as described in [21].

### 2.5. 3’-5’ Exonuclease Assay

The incubation mixture contained, in a final volume of 12.5 µL, 50 mM Tris-HCl, pH 7.5, 1 mM DTT, 4% (*v/v*) glycerol, 0.1 mg/mL BSA, 1.2 nM of either 5’-labeled sp1/sp1c+6 or sp1, 10 mM MgCl_2_, and 5 nM of either the wild-type or the indicated Phi29 DNA polymerase variant. Samples were incubated at 25 °C for the indicated times, and the reaction quenched by adding EDTA up to a final concentration of 10 mM. Reaction products were analyzed by electrophoresis in 7 M urea-20% polyacrylamide gels and autoradiography. Total degradation was obtained by calculating the number of catalytic events giving rise to each degradation product. From these data, the catalytic efficiency of each mutant derivative was calculated relative to the wild-type Phi29 DNA polymerase.

### 2.6. DNA Gel Retardation Assay

The interaction of either the wild-type or the Phi29 DNA polymerase mutants with the primer/template structure was assayed using the 5’-labeled 15mer/21mer hybrid as substrate. The incubation mixture contained, in a final volume of 20 µL, 12 mM Tris-HCl, pH 7.5, 1 mM EDTA, 20 mM ammonium sulphate, 0.1 mg/mL BSA, 10 mM MgCl_2_, 0.7 nM of sp1/sp1c+6 (15mer/21mer) DNA molecule, and the specified amount of either the wild-type or the indicated mutant Phi29 DNA polymerase. After incubation for 5 min at 4 °C, samples were subjected to electrophoresis in precooled 4% (*w/v*) polyacrylamide gels (acrylamide/bis-acrylamide 80:1, *w/w*) containing 12 mM Tris-acetate, pH 7.5 and 1 mM EDTA, and run at 4 °C in the same buffer at 8 V/cm [22]. After autoradiography, Phi29 DNA polymerase/DNA stable interaction was detected as a shift (retardation) in the migrating position of the labeled DNA and quantified by densitometry of the autoradiograms corresponding to different experiments.

### 2.7. Replication of Primed M13 DNA

The incubation mixture contained, in 25 μL, 50 mM Tris–HCl pH 7.5, 10 mM MgCl_2_, 1 mM DTT, 4% (*v/v*) glycerol, 0.1 mg/mL of BSA, 40 μM dNTPs and [α-^32^P] dATP (0.5 μCi), 0.05% (*v/v*) Tween 20, 4.2 nM of primed M13mp8 ssDNA, and 120 nM of either the wild-type or the indicated mutant Phi29 DNA polymerase. Samples were incubated for the indicated times at 30 °C, and the reactions were stopped by adding 20 mM EDTA, 0.3% dodecyl sulfate sodium (SDS), and 0.2 M NaCl. The labeled DNA was denatured by treatment with 0.7 M NaOH and subjected to electrophoresis in alkaline 0.7% agarose gels, as described [23]. After electrophoresis, unit-length M13mp8 ssDNA was detected by ethidium bromide staining, and then, gels were dried and autoradiographed.

### 2.8. Processivity Assay

The incubation mixture contained, in 12.5 μL, 50 mM Tris-HCl, pH 7.5, 10 mM MgCl_2_, 1 mM DTT, 4% (*v/v*) glycerol, 0.1 mg/mL of BSA, 100 nM dNTPs, 1.2 nM 5’-labeled 15/28 mer, and the specified amounts of either the wild-type or the indicated DNA polymerase mutant. Samples were incubated for 5 min at 25 °C, and reactions stopped by adding EDTA to 10 mM. Samples were analyzed by electrophoresis in 7 M urea- 20% polyacrylamide gels and autoradiography.

### 2.9. Polymerization Activity under Single Binding Conditions

The incubation mixture contained, in 12.5 μL, 50 mM Tris-HCl, pH 7.5, 1 mM DTT, 4% (*v/v*) glycerol, 0.1 mg/mL of BSA, 100 nM dNTPs, 1.2 nM 5’-labeled 15/28 mer, and the indicated concentration of either the wild-type, the specified Phi29 DNA polymerase mutant, or the Klenow fragment of *E. coli* DNA polymerase I. The mixture was preincubated for 10 min at 4 °C to allow the formation of the DNA polymerase/DNA complex. The reaction was initiated by the simultaneous addition of 10 mM MgCl_2_ and 1600-fold excess of calf-thymus activated-DNA as a challenger DNA. Samples were incubated 2.5 min at 25 °C. As a control of the trapping efficiency of the challenger DNA, parallel reactions were carried out preincubating the DNA polymerase simultaneously with the labeled primer/terminus molecule and the trapping DNA. In these cases, the reaction was initiated by adding 10 mM MgCl_2_. The reactions were stopped by adding EDTA to 10 mM. Samples were analyzed by electrophoresis in 7 M urea- 20% polyacrylamide gels and autoradiography.

### 2.10. Protein-Primed Initiation Assay (Terminal Protein-Deoxyadenosine Monophosphate Formation)

The ability to carry out the initiation step during TP-DNA replication was analyzed as described [24]. The incubation mixture contained, in 25 μL, 50 mM Tris-HCl, pH 7.5, 10 mM MgCl_2_, 20 mM ammonium sulphate, 1 mM DTT, 4% (*v/v*) glycerol, 1.6 nM of Phi29 TP-DNA as template, 0.1 mg/mL BSA, 0.2 μM dATP (1μCi [α- ^32^P]dATP), 19 nM of either wild-type or mutant DNA polymerase, and 19 nM of TP. Samples were incubated for 1, 2, or 4 min at 30 °C. Reactions were stopped by adding 10 mM EDTA-0.1% SDS, and the samples were filtered through Sephadex G-50 spin columns and further analyzed by SDS-polyacrylamide gel electrophoresis (PAGE) in 12% polyacrylamide gels. Quantification was done by densitometric analysis of the labeled band corresponding to the TP-dAMP complex detected by autoradiography (Agfa Healthcare, Belgium).

### 2.11. Interference Assay for Terminal Protein Binding

The assay was performed essentially as described in [24]. Reactions were carried out as explained for the TP-dAMP formation, but without template, using a limited amount of TP (15 nM), a fixed amount of either the wild-type or the indicated mutant DNA polymerase, and increasing amounts of the DNA polymerase mutant D249E [25] (0–120 nM) that is catalytically inactive but conserves, while intact, the capacity to interact with the TP, and 1mM MnCl_2_. In all cases, the incubation was for three hours at 30 **°**C. After incubation, reactions were stopped and analyzed, as indicated for the protein-primed initiation assay.

### 2.12. Analysis of the Interaction between Terminal Protein and DNA Polymerase Mutants by Glycerol-Gradient Ultracentrifugation

The assay was performed essentially as described in [24]. The incubation mixture contained, in 150 μL, 50 mM Tris–HCl, pH 7.5, 1 mM DTT, 0.1 mg/mL BSA, 20 mM ammonium sulphate, 0.6 μM of either the wild-type or the indicated mutant DNA polymerase, and 0.6 μM of TP. After incubation for 30 min at 4 °C, samples were loaded on top of a continuous 15–30% (*v/v*) glycerol gradient (4 mL) in the presence of 50 mM Tris-HCl, pH 7.5, 20 mM ammonium sulphate, 0.2 M NaCl, 1 mM EDTA and 7 mM β-mercaptoethanol, and centrifuged at 4 °C for 24 h at 58000 rpm in a Beckman TST 60.4 rotor. Gradients were fractionated and subjected to SDS-12% polyacrylamide gel electrophoresis. The proteins in the gel were stained with Coomasie blue to identify the peaks corresponding to the TP/DNA polymerase heterodimer (97 kDa) and the free monomers of TP (31 kDa) and DNA polymerase (66 kDa).

### 2.13. Terminal Protein-DNA Replication Assay

The assay was performed essentially as described in [24], in the presence of 50 mM Tris-HCl, pH 7.5, 10 mM MgCl_2_, 20 mM ammonium sulphate, 1mM DTT, 4% glycerol, 0.1 mg/mL BSA, 20 μM each dNTP and [α-^32^P]dATP (1 μCi), 16 nM of Phi29 TP-DNA, 19 nM of either the wild-type DNA polymerase or the indicated mutant, and 19 nM of TP in a final reaction volume of 25 μL. After incubation for the indicated times at 30 °C, the reaction was stopped by adding 10 mM EDTA-0.1% SDS, and the samples were filtered through Sephadex G-50 spin columns (Sigma, San Luis, MO, USA). Quantification of the DNA synthesized in vitro was carried out from the Cerenkov radiation corresponding to the excluded volume. The labeled DNA was denatured by treatment with 0.7 M NaOH and subjected to electrophoresis in alkaline 0.7% agarose gels, as described [23]. After electrophoresis, the position of unit length Phi29 DNA was detected by ethidium bromide staining, and then, the gels were dried and autoradiographed.

## 3. Results

### 3.1. Site-Directed Mutagenesis at Terminal Protein Region 1 Phi29 DNA Polymerase Residues

To analyze the role of the mentioned residues belonging to the TPR1 loop of Phi29 DNA polymerase, we changed them into alanine in a non-conservative change, obtaining the DNA polymerase variants R306A, R308A, F309A, Y310A, and K311A. We also obtained the deletion mutant Δ6 lacking residues Arg306-Lys311. The mutant derivatives were overexpressed and purified as described in Materials and Methods, and their activity was analyzed by in vitro biochemical assays.

### 3.2. Changes at Phi29 DNA Polymerase TPR1 Loop Residues Impair the Stabilization of the DNA Primer Terminus at the Polymerization Active Site

**Figure 2:**
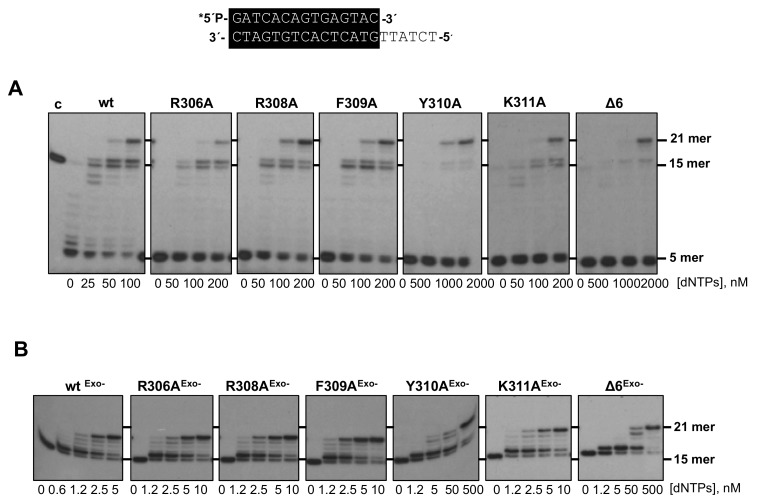
(**A**) DNA polymerase/exonuclease coupled assay. The assay was performed as described in Materials and Methods using the 5’-labeled primer/template molecule 15mer/21mer depicted on top of the figure and the indicated concentration of dNTPs. Asterisk indicates the ^32^P 5’-labeled end of the primer strand. Polymerization or 3’–5’ exonucleolysis is detected as an increase or decrease, respectively, in the size of the primer (15mer). (**B**) DNA polymerization catalyzed by wild-type^Exo-^ (D12A/D66A) [18], R306A^Exo-^, R308A^Exo-^, F309A^Exo-^, Y310A^Exo-^ (Y310A/D12A/D66A), K311A^Exo-^, and Δ6^Exo-^ DNA polymerases. The assay was performed as described in (**A**), using the 5’-labeled molecule 15mer/21mer and the indicated concentration of dNTPs as substrate; c: control DNA.

As most DNA-dependent DNA polymerases, the exonuclease and polymerization activities of Phi29 DNA polymerase reside in the structurally independent N-terminal and C-terminal domains, respectively [10,26]. Despite their structural separation, both active sites must work in concert to ensure a productive and accurate replication reaction, preventing the accumulation of errors in the newly synthesized strand while allowing a proper elongation rate [27]. The decision to synthesize versus to degrade the primer-terminus depends on several factors, such as the catalytic rate of both activities and the comparative stabilization of the primer-terminus at such active sites [28,29]. To evaluate how the mutations introduced affected the dynamic equilibrium between the 3’–5’ exonuclease and polymerization activities of the DNA polymerase, we studied the functional coupling between synthesis and degradation on a primer/template hybrid molecule (sp1/sp1c+6) as a function of dNTP concentration (see Materials and Methods). Without nucleotides, the only bands detected would correspond to the exonucleolytic degradation of the primer, allowing us to evaluate whether the introduced changes alter the 3’–5’ exonuclease activity of the enzyme. As the concentration of the provided dNTPs increases, the exonuclease activity is progressively competed by the 5’–3’ polymerization one. Net deoxynucleosides monophosphate (dNMPs) incorporation is observed as an increase in the size of the labeled primer, allowing us to define the dNTPs concentration needed to obtain an efficient elongation for each mutant derivative (Pol/Exo ratio). As shown in Figure 2A, except for the Y310A and Δ6 variants, the rest of the mutants, although able to entirely replicate the protruding template reaching the 21mer position, needed a dNTPs concentration 2-fold higher (200 nM) than the wild-type enzyme (100 nM). In the case of mutants Y310A and Δ6, they required 1 µM and 2 µM, respectively, to get a primer elongation efficiency similar to that obtained with the wild-type polymerase. Importantly, the 3’–5’ exonuclease activity of the mutant enzymes was higher than that of the wild-type (Figure 2A, lanes without nucleotides).

To discern if the unbalanced equilibrium displayed by the mutants was due to their higher exonucleolysis or to a defect in the polymerization activity, the mutants were engineered to include the double mutation D12A/D66A [18] at two catalytic residues of the exonuclease active site to eliminate their exonuclease activity (R306A^Exo-^, R308A^Exo-^, F309A^Exo-^, Y310A^Exo-^, and K311A^Exo-^ and Δ6^Exo-^). As it is shown in Figure 2B, exonuclease deficient DNA polymerase mutants D12A/D66A (wt^Exo-^, used as control of a wild-type polymerization active site), R306A^Exo-^, R308A^Exo-^, F309A^Exo-^, and K311A^Exo-^ required a 2.5–5 nM dNTPs to reach the 21mer position, whereas mutants Y310A^Exo-^ and Δ6^Exo-^ needed a much higher concentration (50 nM) to fulfill replication of the template strand. Therefore, the polymerization defects observed in these two mutants cannot be attributed to a higher exonucleolytic activity but to impaired polymerization catalysis.

**Figure 3:**
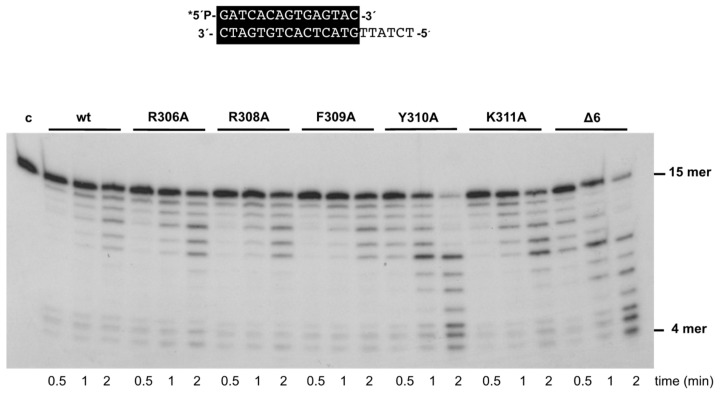
The 3’–5’ exonuclease activity of Phi29 DNA polymerase mutants. The assay was performed as described in Materials and Methods, using the 5’-labeled primer/template molecule 15mer/21mer depicted on top of the figure. After incubation for the indicated times at 25 °C, degradation of the labeled DNA was analyzed by electrophoresis in 7M urea-20% polyacrylamide gels and autoradiography. The position of the 4mer degradation intermediate of the sp1 substrate (15mer) is indicated. Asterisk indicates the 5’-^32^P-labeled end of the primer strand. c: control DNA.

**Figure 4:**
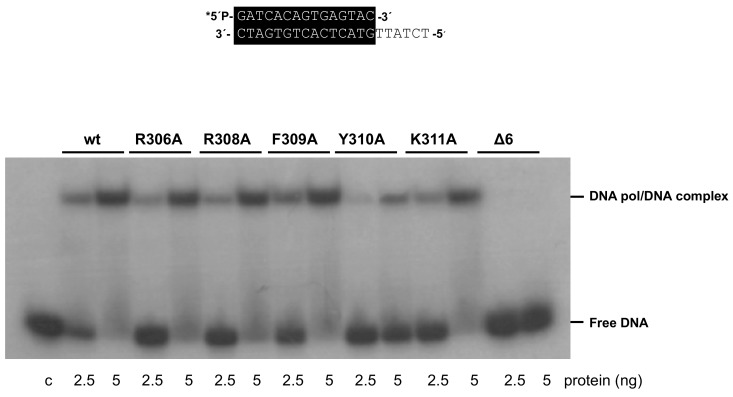
Gel retardation of dsDNA by wild-type and mutant Phi29 DNA polymerases. The assay was carried out as described in Materials and Methods, using the 5’-labeled primer/template molecule 15mer/21mer depicted on top of the figure, in the presence of the indicated amounts of enzyme. After non-denaturing gel electrophoresis, the bands corresponding to free dsDNA and to the DNA polymerase-DNA complex were detected by autoradiography. Asterisk indicates the 5’-^32^P-labeled end of the primer strand; c: control DNA.

As mentioned above, the 3’–5’ exonuclease activity of the mutant enzymes was apparently higher than that of the wild-type enzyme under those experimental conditions. To study this activity in depth, we performed time-course experiments (see Materials and Methods) using the primer/template molecule as the substrate of the exonuclease activity. As shown in Figure 3 and Table 1, the activity of mutants R306A, R308A, F309A, and K311A was similar to that of the wild-type enzyme, whereas that of mutants Y310A and Δ6 was 1.6- and 1.3-fold higher. Importantly, these variants exhibited a nearly wild-type exonuclease activity on an ssDNA substrate (see Supplementary Figure S1A). In addition, the wild-type and the mutant polymerases hydrolyzed, at a comparable rate, the minimal substrate 5’-*p-*nitrophenyl ester of thymidine 5’-monophosphate (*p*NP-TMP; see Materials and Methods and Supplementary Figure S1B), in which the binding at the exonuclease active site is based only on the ligands responsible for the stabilization of the 3’ end of an ssDNA [21], reflecting the integrity of their 3’–5’ exonuclease active site. Altogether, the defects in the polymerization reaction and the higher proficiency of the exonuclease activity of mutants Y310A and Δ6, specifically on a primer/template substrate, could be pointing to a defective stabilization of the primer terminus at their polymerization active site. To test this hypothesis, we analyzed the capacity of the mutant derivatives to efficiently bind a primer-terminus by gel shift assays (as described in Materials and Methods). As shown in Figure 4, the wild-type enzyme gave rise to a single retardation band, that has been interpreted as a stable complex competent for polymerization, in which the primer-terminus is stabilized at the polymerization active site [30]. Mutants displayed a wild-type behavior, with the exception of Δ6, and to a lower extent Y310A, which were impaired in binding dsDNA (7% and 29%, respectively; Figure 4 and Table 1). These results suggest a defective stabilization of the primer terminus at the polymerization active site of mutants Y310A and Δ6, which could account for both their polymerization defects and improved exonuclease activity, due to preferential binding of the primer terminus at the exo site.

### 3.3. Processive DNA Synthesis Coupled to Strand Displacement by Phi29 DNA Polymerase Variants

**Figure 5:**
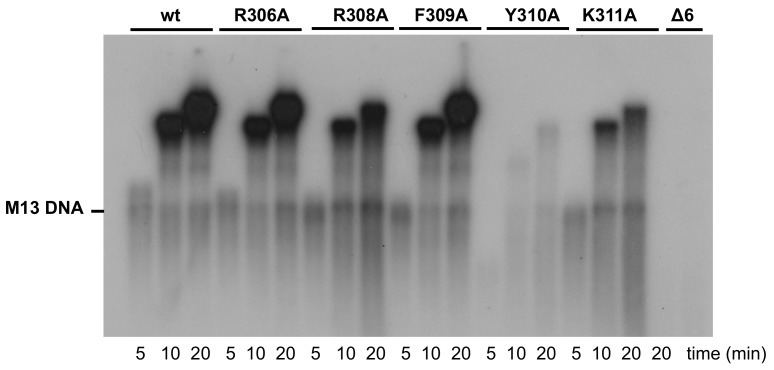
Strand-displacement coupled to M13 DNA replication by wild-type and mutant DNA polymerases. Replication of primed-M13 DNA was carried out as described in Materials and Methods. After incubation for the indicated times at 30 °C, relative activity values were calculated from dNMP incorporation. The position of unit-length M13 DNA is shown on the left.

The most distinctive feature exhibited by Phi29 DNA polymerase is its ability to couple processive DNA synthesis to strand displacement, a unique property that allows the enzyme to perform the viral replication from a single DNA binding event in the absence of accessory processivity factors and helicases [9]. Thus, to analyze whether the changes introduced in the DNA polymerase have an effect in processive DNA synthesis coupled to strand displacement, we performed a DNA-primed M13 replication assay (see Materials and Methods), wherein an oligonucleotide was hybridized to the circular M13 ssDNA molecule, and the Phi29 DNA polymerase starts DNA replication from the 3’-OH end of the hybridized oligonucleotide. The first replication round does not require strand displacement, but when it is completed, the polymerase encounters the 5’ end of the primer, requiring active strand displacement to continue DNA synthesis. As shown in Figure 5 and Table 1, except for mutant Δ6 that did not give rise to detectable replication products, the rest of the polymerase derivatives were able to couple processive replication to strand displacement as they yielded replication products longer than the unit length (7 kb). Whereas the replication efficiency displayed by mutants R306A and F309A was similar to that of the wild-type enzyme (92% and 87%, respectively), mutants R308A, Y310A, and K311A replicated the M13 ssDNA 3-, 33-, and 6-fold less efficiently. In the case of mutant Y310A, it also exhibited an impaired replication rate, as deduced from the shorter length of the synthesized products, a fact that could be suggesting a defective processivity. Therefore, to analyze if the processivity of the mutants was affected, we evaluated the chain length distributions during DNA polymerization as a function of the enzyme/DNA ratio. As shown in Supplementary Figure S2, the progressive decrease of the enzyme/DNA ratio did not have any impact on the length of the replication products by the wild-type enzyme, as expected for a processive DNA polymerase. As it can be observed, mutants R306A, R308A, F309A, and K311A also exhibited a processive replication pattern. The high exonuclease activity of mutants Y310A and Δ6, together with their polymerization defects (see above), precluded the evaluation of their processivity under those conditions. Thus, we conducted similar experiments but using the exonuclease deficient versions (Y310A^Exo-^ and Δ6^Exo-^). As shown in Supplementary Figure S3A, both mutants seemed to be slightly affected in the processivity. In fact, in the presence of an excess of activated DNA acting as a challenger, they were able to incorporate only the first nucleotide due to a prompt dissociation of the DNA (Supplementary Figure S3B).

### 3.4. Mutations at Phi29 DNA Polymerase Terminal Protein Region 1 Loop Affect the Interaction with the Terminal Protein

**Figure 6:**
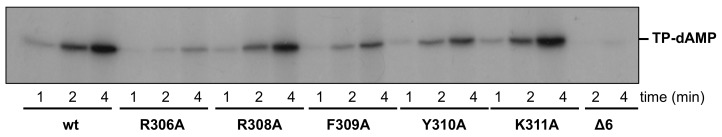
In vitro protein-primed initiation. The initiation reaction was performed as described in Materials and Methods, using as template Phi29 TP-DNA. After incubation for at the indicated times at 30 °C, the reaction was stopped, processed, and analyzed by dodecyl sulfate sodium-polyacrylamide gel electrophoresis (SDS-PAGE) and autoradiography. The position of the terminal protein-deoxyadenosine monophosphate (TP-dAMP) complex is indicated.

As mentioned above, to start Phi29 DNA replication, the DNA polymerase has to form a heterodimer with a free TP, then the DNA polymerase catalyzes the incorporation of the first nucleotide onto the hydroxyl group of the TP priming residue Ser232 (initiation reaction). Thus, to study the initiation activity of the mutant polymerases, we evaluated their ability to form the initiation product (TP-dAMP) using as template Phi29 TP-DNA. As shown in Figure 6 and Table 1, mutant K311A and, to a lower extent, R308A, were proficient in performing the initiation reaction, whereas mutants R306A, F309A, and Y310A were 13-, 4- and 3-fold less efficient than the wild-type enzyme. As it can be observed, mutant Δ6 showed a negligible ability to catalyze the formation of the initiation product.

**Figure 7:**
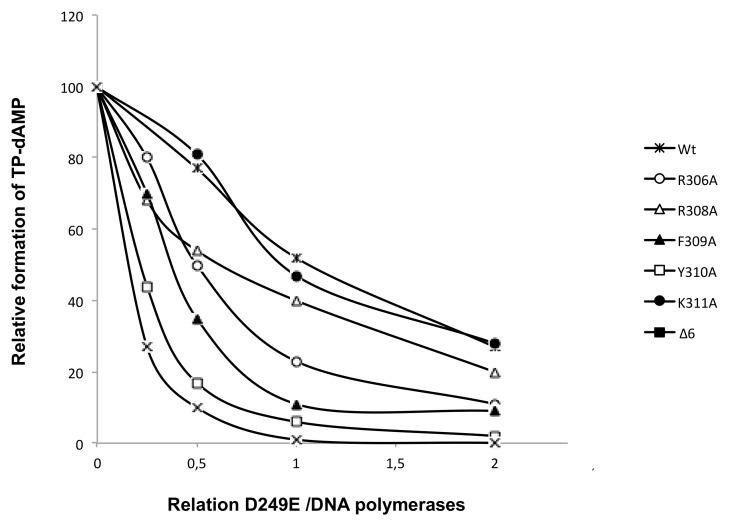
Competition for TP between D249E [25] and DNA polymerase mutants. The assay of formation of TP-dAMP was performed, as described in Materials and Methods, in the presence of a limited amount of TP, a fixed amount of wild-type and mutant DNA polymerase, and increasing amounts of the DNA polymerase mutant D249E. Reactions were started by adding 1 mM MnCl_2_, and after incubation for three hours at 30 °C, were stopped and analyzed as indicated for the protein-primed initiation assay. The TP-dAMP formed in the different competition conditions relative to that formed in the absence of competition (100%) is indicated.

To evaluate if the diminished TP-dAMP complex formation displayed by the mutant enzymes was due to a decreased ability to interact with the TP, we performed interference assays where the wild-type and mutant DNA polymerases compete for a limiting amount of TP with the DNA polymerase mutant D249E that is catalytically inactive but conserves an intact capacity to interact with the TP [25]. As shown in Figure 7, and as expected, when the D249E/wild-type ratio was 1, the formation of the TP-dAMP product was reduced 2-fold. A similar result was obtained with mutants R308A and K311A. Conversely, the initiation reaction displayed by mutants R306A, F309A, Y310A, and Δ6 decreased to less than 50% with respect to that exhibited in the absence of the competitor D249E mutant, a result that indicates an impaired interaction of those mutants with the TP, in agreement with the results obtained in the initiation assays.

**Figure 8:**
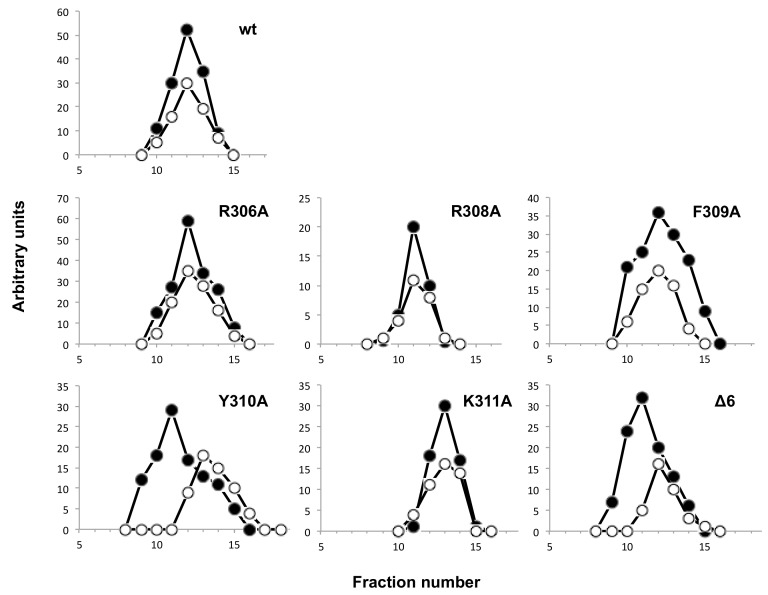
Analysis of TP/DNA polymerase interaction by glycerol gradient ultracentrifugation. The assay was carried out as described under Materials and Methods, preincubating 0.6 μM of wild-type TP with 0.6 μM of either the wild-type or the indicated mutant DNA polymerase. After incubation for 30 min at 4 °C, samples were loaded on top of a continuous 15–30% glycerol gradient in the presence of 0.2 M NaCl. After centrifugation, the collected fractions were subjected to SDS-12% polyacrylamide gel electrophoresis and further stained with Coomasie. Densitometric quantification, expressed in arbitrary units, of both DNA polymerase (full circles) and TP (open circles), is represented.

The DNA polymerase/TP interaction was also directly analyzed by glycerol gradient ultracentrifugation (see Materials and Methods). As shown in Figure 8, except for mutants Y310A and Δ6, the wild-type polymerase and the rest of mutant derivatives formed a stable heterodimer with the TP, both proteins sedimenting in the same fractions. Conversely, mutants Y310A and Δ6 and TP eluted as monomers, indicating a deficient TP binding. The low interference ability exhibited by mutants R306A and F309A, together with their capacity to form a stable heterodimer, is suggestive of a non-functional interaction of these mutants with the TP. These results, together with the fact that the mutants Y310A and Δ6 were also the ones most affected in binding the dsDNA, indicate a role of the TPR1 loop and, specifically of the residue Tyr310 not only in binding the DNA but also the TP, the natural primer of the polymerase.

**Figure 9:**
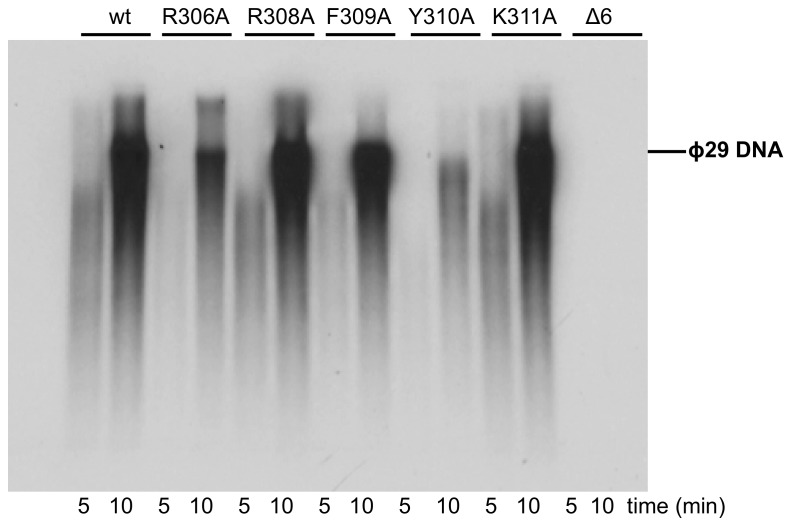
TP-DNA replication. The assay was carried out as described in Materials and Methods. After incubation for the indicated times at 30 °C, relative values were calculated (Table 1), and the length of the synthesized DNA was analyzed by alkaline agarose gel electrophoresis. The migration position of unit-length Phi29 DNA is indicated.

Once catalyzed the formation of the TP-dAMP product, the same DNA polymerase molecule elongates it via strand displacement to produce full-length Phi29 TP-DNA [9]. Thus, replication assays were carried out using a minimal replication system based on Phi29 TP-DNA, DNA polymerase, and TP [9]. As it can be observed in Figure 9 and Table 1, mutants R308A, F309A, and K311A were moderately affected, whereas mutant R306A and particularly Y310A were severely impaired in the replication reaction, in agreement with their hindered initiation activity. As expected, the deletion of the TPR1 loop in mutant Δ6 precluded the replication of TP-DNA.

## 4. Discussion

Bacteriophage Phi29 DNA polymerase belongs to the B family of DNA polymerases and is the enzyme responsible for the viral DNA replication [31]. This enzyme is the only member of the protein-primed subgroup of DNA polymerases in which the structure has been solved [10]. It consists of an N-terminal domain with the exonuclease activity and a C-terminal domain with the polymerization activity. It also has two insertions specifically present in the DNA polymerases that initiate replication by a protein-priming mechanism: TPR1 (terminal protein region 1) and TPR2 (terminal protein region 2). Previous crystallographic and biochemical studies showed residues of the TPR1 subdomain forming a network of interactions with the TP [11,13], as the salt bridges between DNA polymerase: TP residues Glu322: Arg169 and Glu291: Arg158, required to maintain the stability of the heterodimer and the proper positioning of the priming-domain at the polymerization site [16,32]. In addition, by swapping of both the C-terminal priming domain of the related Phi29 and GA-1 TPs and the TPR1 subdomain of the cognate DNA polymerase, allowed us to conclude that the interaction between TPR1 and the intermediate domain of TP is the one mainly responsible for the specificity between both proteins [12].

The comparison of the structure of the Phi29 DNA polymerase forming a heterodimer with the TP and the apo enzyme, showed that the main difference is a conformational change in some residues of TPR1 (304–314) that are forming a loop with a high degree of flexibility in the apo enzyme. This loop moves to allow TP access to the active site of the polymerase [16].

Previous studies proposed a model in which the TP intermediate domain would recognize and interact with the DNA polymerase TPR1 subdomain, promoting the TPR1 loop change from a flexible to stable moved-out conformation, that would now allow the proper placement of the TP priming domain into the DNA polymerase structure [12]. In order to further study the role of this TPR1 loop, we have mutated the electropositive and aromatic residues of the TPR1 loop into alanine, as well as obtained a deletion mutant lacking the TPR1 loop.

The Phi29 DNA polymerase mutants of the TPR1 loop (particularly Y310A and Δ6) exhibited a 3’–5’ exonuclease activity higher than that of the wild-type enzyme and an impaired polymerization reaction that shifted the exo/pol equilibrium towards primer degradation. Both the increased exonuclease and the reduced polymerization could be caused by the defective stabilization of the primer-terminus at the polymerization active site, as deduced from the gel retardation assays. These results parallel those obtained with changes introduced at residues Lys305 and Tyr315, also belonging to the TPR1 subdomain, and at residues from the KxY [33] and Tx_2_G/AR [30] motifs of the C-terminal polymerization domain of Phi29 DNA polymerase, as the mutations introduced also provoked a decrease of the DNA binding stability that led to an increased exonucleolytic activity and a concomitant reduction of the synthetic activities. Therefore, both motifs, together with the TPR1 subdomain, would be responsible for the stabilization of the primer terminus at the polymerization active site.

**Figure 10:**
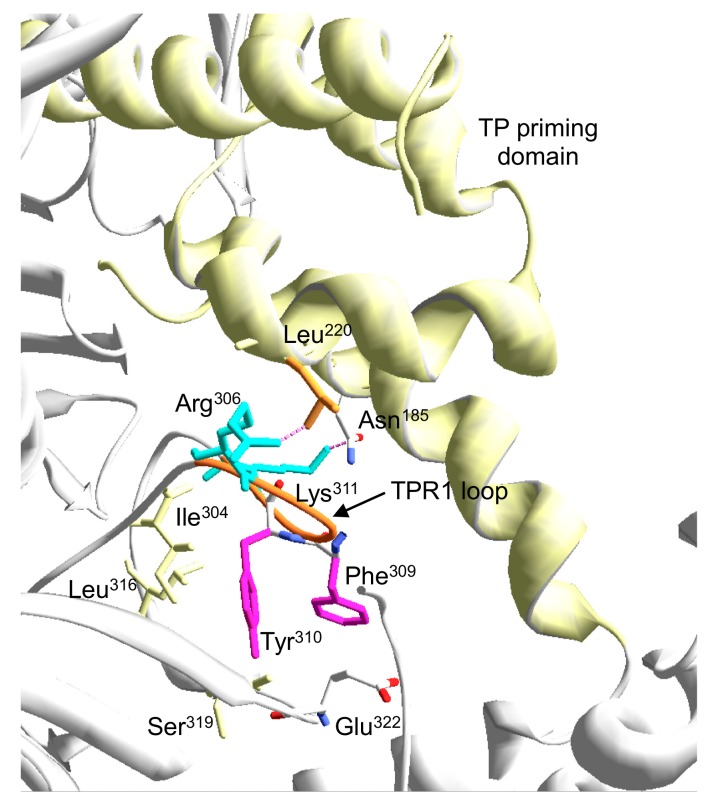
Detailed view of the interaction of TPR1 loop residues with the TP priming domain. The electropositive and aromatic residues of the TPR1 loop studied here are shown in cyan and magenta sticks, respectively. Flexible TPR1 loop regions (residues 305–315) are colored in orange. Terminal protein priming domain is shown in ribbon representation (colored in yellow). For details, see main text.

The defects in replicating Phi29 TP-DNA exhibited by mutants R306A, F309A, Y310A, and Δ6 were mainly due to an inefficient initiation reaction caused by a defective interaction with the primer TP, as deduced from the interference and glycerol gradient sedimentation assays. Mutants Y310A and Δ6 also displayed an additional impairment in stabilizing the DNA primer-terminus at the polymerization active site, dramatically reducing their ability to fulfill Phi29 TP-DNA replication. These results point to a pivotal role of Tyr310 in the proper placement of the DNA and TP 3’-OH termini at the polymerization site during the elongation and initiation of DNA replication. The crystallographic structure of the Phi29 heterodimer shows that Tyr310 is packed against the TPR1 residues Ile304, Leu316, and Ser319 (see Figure 10). This network of interactions could be significant for making possible the movement of the TPR1 loop required to allow the access of both DNA and TP-priming domain to the polymerization active site. In addition, the fact that the polymerization activity of mutants R306A and F309A increases significantly on DNA substrates as primed M13 and primer/template hybrids, suggests a more specific role for these residues in the stabilization of the TP priming domain at the catalytic site. Although the mutant enzymes formed a stable heterodimer with the TP, their hindered ability to compete with DNA polymerase mutant D249E in the interference assays could be indicative of a stable but non-functional interaction with the TP that precludes the proper positioning of the TP-priming loop at the preinsertion site of the polymerase. The crystallographic structure of the Phi29 DNA polymerase/TP heterodimer showed an ionic interaction between the TPR1 residue Glu322 and the Arg169 located at the intermediate domain of the TP [16]. Biochemical analysis of Phi29 DNA polymerase mutant R169A [32] demonstrated the critical role of this interaction in the priming TP reactions. In the heterodimer, a structure could be observed in which Phe309 stacks against Glu322. The phenotype of mutant F309A leads us to propose a role for Phe309 residue in both stabilizing the TPR1 loop conformation to allow the access of the TP priming domain to the polymerization site and in guaranteeing the formation of a functional complex between the DNA polymerase and the TP through the Glu322-Arg169 interaction [32]. In addition, the crystallographic structure of the heterodimer shows a potential contact between residue Arg306 and the TP priming domain residue Leu220 (see Figure 10) that would contribute to the proper interaction between both proteins during the initiation reaction, so that the loss of such an interaction would account for the specific impairment of the initiation reaction exhibited by mutant R306A.

## 5. Conclusions

Our results support a pivotal role for the TPR1 subdomain loop in the proper stabilization of the Phi29 DNA polymerase primer molecules (DNA and TP) at the polymerization active site, specifically through residues Arg306 and Tyr310, to allow efficient initiation and further elongation steps during viral genome replication.

## Figures and Tables

**Table 1:** Enzymatic activities of wild-type and mutant Phi29 DNA polymerases.

Phi29 DNA Polymerase Mutants								
Assay	Substrate	Wild-Type	R306A	R308A	F309A	Y310A	K311A	Δ6
Exonuclease	dsDNA	100	106 ± 11	99 ± 10	90 ± 6	164 ± 11	108 ± 7	131 ± 9
Retarding	dsDNA	100	82 ± 23	113 ± 49	89 ± 34	29 ± 6	92 ± 41	7 ± 4
Replication	M13-DNA	100	92 ± 4	39 ± 3	87 ± 6	3 ± 1	18 ± 1	n.d.
Initiation	TP-DNA	100	8 ± 1	49 ± 9	25 ± 2	30 ± 2	100 ± 19	3 ± 1
Replication	TP-DNA	100	20 ± 6	57 ± 16	34 ± 12	11 ± 3	73 ± 23	n.d.

Data represent the mean value and the standard deviation obtained from at least three independent experiments. Numbers indicate the activity of mutant DNA polymerases with respect to the wild-type enzyme; n.d., not detected.

## Supplementary Materials

The following are available online at https://www.mdpi.com/2218-273X/9/11/648/s1, Figure S1: 3’–5’ exonuclease activity of Phi29 DNA polymerase mutants, Figure S2: Processivity assay of Phi29 DNA polymerase mutants, Figure S3: Processivity assay of Phi29 DNA polymerase variants wild-type^Exo-^, Y310A^Exo-^, and Δ6^Exo-^.
